# Supplementary material for: A unified method to revoke the private data of patients in intelligent healthcare with audit to forget
Source: Nat Commun. 2023 Oct 6;14:6255. doi: 10.1038/s41467-023-41703-x (PMC10558551; doi:10.1038/s41467-023-41703-x)
Supplement: Supplementary file 1 — Supplementary Information [file 41467_2023_41703_MOESM1_ESM.pdf]

Supplementary information for
A Unified Method to Revoke the Private Data of Patients in Intelligent Healthcare with Audit to Forget

Juexiao Zhou[1,2,#], Haoyang Li[1,2,#], Xingyu Liao[1,2], Bin Zhang[1,2], Wenjia He[1,2], Zhongxiao Li[1,2], Longxi Zhou[1,2], Xin Gao[1,2,*]

[1]*Computer Science Program, Computer, Electrical and Mathematical Sciences and Engineering Division, King Abdullah University of Science and Technology (KAUST), Thuwal 23955-6900, Kingdom of Saudi Arabia*
[2]*Computational Bioscience Research Center, King Abdullah University of Science and Technology, Thuwal 23955-6900, Kingdom of Saudi Arabia*
[*]*Corresponding author*
[#]*Equal contribution*

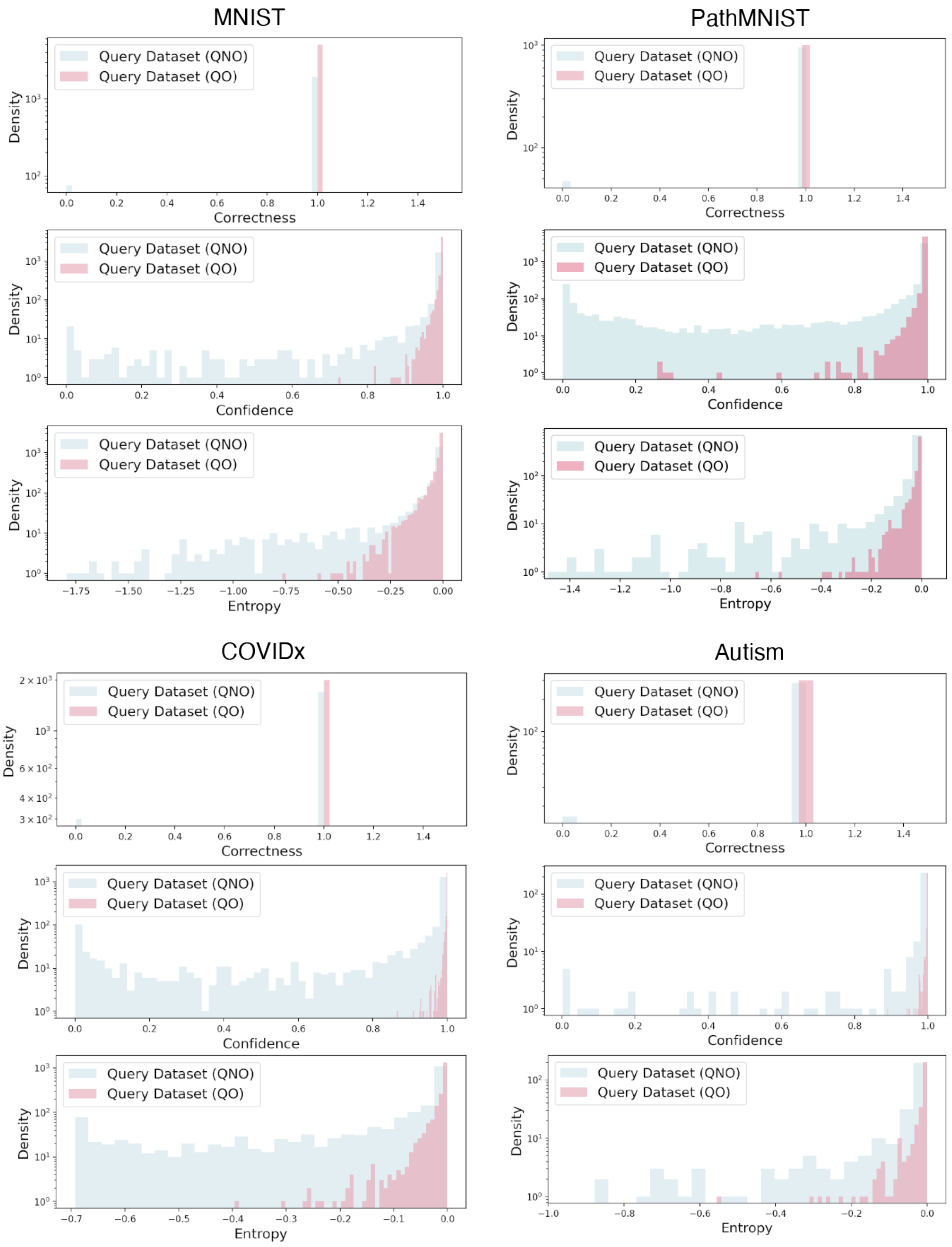

Fig. S1. Distribution of correctness, confidence, and entropy of QO and QNO on four datasets. The four datasets are MNIST, PathMNIST, COVIDx, and Autism.

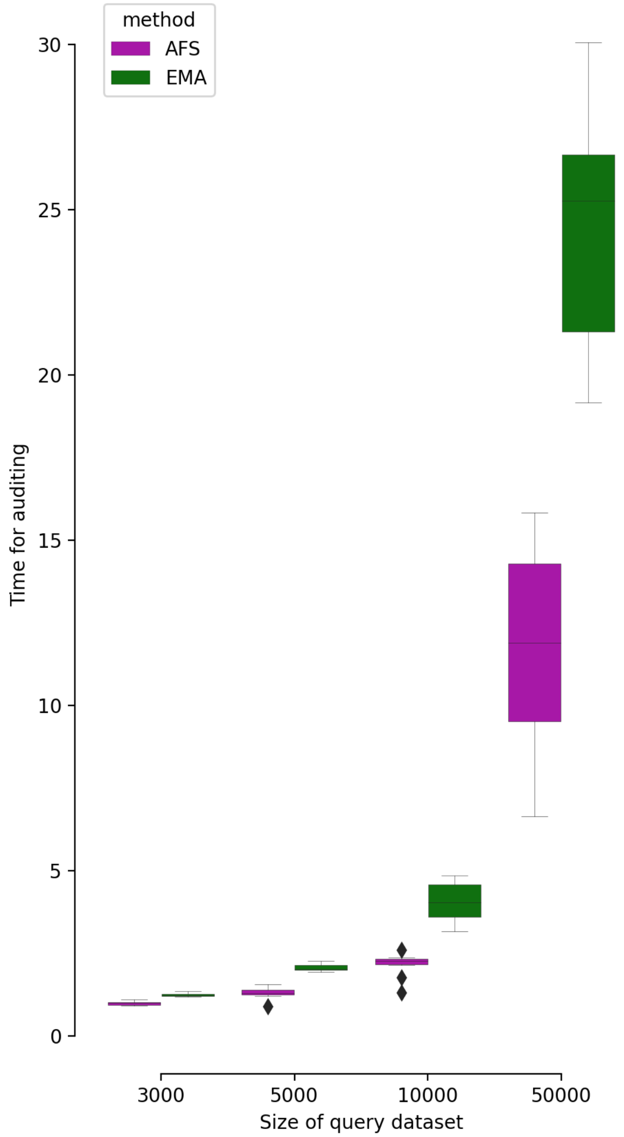

Fig. S2. Time for auditing of AFS and EMA when varying the size of the query dataset. The x-axis represents the size of the query dataset and the y-axis represents the time for auditing in seconds.

Table S1. Comparison of inference times. Time for inferring 100 samples with the model retrained sith SISA, the model retrained with CF-k/EU-k, the original model and the model generated by AFS.

Dataset	SISA (10 shards)	CF-k/EU-k	Original model	New model generated by AFS
MNIST	$438\mu s \pm 1.28\mu s$	$439\mu s \pm 1.41\mu s$	439 $\mu s \pm 1.54\mu s$	284 $\mu s \pm 447ns$
PathMNIST	$5.13ms \pm 17\mu s$	$5.14ms \pm 10\mu s$	5.13 ms $\pm 22\mu s$	4.99 ms $\pm 14.1\mu s$
COVIDx	$1.27s \pm 355ms$	$1.28s \pm 634ms$	1.27 s $\pm 500ms$	661 ms $\pm 9.98ms$
Autism	$126\mu s \pm 213ns$	$126\mu s \pm 302ns$	126 $\mu s \pm 177ns$	87.3 $\mu s \pm 120ns$

Table S2. Comparison of inference GPU memory. Required GPU memory (MB) for inferring 100 samples with the model retrained sith SISA, the model retrained with CF-k/EU-k, the original model and the model generated by AFS.

Dataset	SISA (10 shards)	CF-k/EU-k	Original model	New model generated by AFS
MNIST	258	258	258	61
PathMNIST	173	173	173	129
COVIDx	17,805	17,805	17,805	10,600
Autism	2	2	2	1
